# Engineered RBC-derived nanovesicles functionalized with tumor-targeting ligands: A comparative study on breast cancer targeting efficiency and biocompatibility

**DOI:** 10.1515/med-2025-1306

**Published:** 2025-10-31

**Authors:** Fulan Yang, Weilun Pan, Jin Jiang, Xingwei Huang, Yue Qiao, Yuan Zhang, Huozhong Yuan, Xin Wang, Bo Li, Jingyun Guo

**Affiliations:** Department of Breast Surgery, Ganzhou Hospital-Nanfang Hospital, Southern Medical University, Ganzhou, Jiangxi Province, 341000, China; Department of Laboratory Medicine, Guangdong Provincial Key Laboratory of Precision Medical Diagnostics, Guangdong Engineering and Technology Research Center for Rapid Diagnostic Biosensors, Guangdong Provincial Key Laboratory of Single-cell and Extracellular Vesicles, Nanfang Hospital, Southern Medical University, Guangzhou, Guangdong Province, 510515, China; Guangzhou Blood Center, The Key Medical Laboratory of Guangzhou, Guangzhou, Guangdong Province, 510095, China; Department of Breast, Ganzhou Cancer Hospital, Ganzhou, Jiangxi Province, 341000, China; Breast Center, Department of General Surgery, Nanfang Hospital, Southern Medical University, Guangzhou, Guangdong Province, 510515, China; The First Department of Breast Cancer, Tianjin Medical University Cancer Institute and Hospital, National Clinical Research Center for Cancer, Tianjin, 300060, China

**Keywords:** red blood cell-derived nanovesicle, tumor targeting, breast cancer, targeting ligands, biomimetic drug delivery

## Abstract

**Introduction:**

Cell membrane-derived nanovesicles, particularly those originating from red blood cells (RNVs), have garnered considerable attention as innovative drug delivery vehicles in oncology, owing to their exceptional biocompatibility, immune evasion, and prolonged systemic circulation. Nevertheless, their inherently poor tumor-targeting efficiency and nonspecific biodistribution present major obstacles to their therapeutic translation.

**Objectives:**

This study sought to functionalize RNVs with a diverse array of tumor-targeting ligands – cRGD, transferrin (TRF), folic acid (FA), GE11, and RVG29 – and to systematically compare their tumor-homing efficiency, biodistribution, and biosafety in a breast cancer model.

**Results:**

Functionalized RNVs exhibited markedly enhanced tumor affinity relative to unmodified vesicles in both *in vitro* and *in vivo* settings. Among the engineered formulations, RNV@cRGD achieved the most pronounced intratumoral accumulation and cellular uptake, followed sequentially by RNV@GE11, RNV@TRF, RNV@FA, and RNV@RVG29. Fluorescence imaging corroborated the superior tumor selectivity of engineered constructs, all of which also demonstrated robust stability and negligible off-target toxicity in murine models.

**Conclusion:**

This work presents systematic comparative evaluation of ligand-engineered RNVs, underscoring cRGD as the most potent targeting moiety for breast cancer. These findings illuminate critical design principles for the rational development of tumor-directed RNV-based drug delivery systems and strengthen the translational promise of biomimetic nanocarriers for clinical oncology.

## 1 Introduction

Cancer remains one of the foremost causes of mortality worldwide, exerting a profound and escalating global burden [1]. According to the most recent GLOBOCAN statistics from the International Agency for Research on Cancer (IARC), breast cancer ranks first in both incidence and mortality among women globally [2]. Despite advances in oncologic management, current therapeutic modalities – including surgery, chemotherapy, radiotherapy, and targeted therapies such as HER2 inhibitors – continue to be hampered by systemic toxicity, drug resistance, and inadequate tumor specificity [3,4]. To overcome these limitations, nanoparticle-based drug delivery systems (DDS) have emerged as promising alternatives, designed to refine the pharmacokinetic and pharmacodynamic profiles of anticancer agents, thereby enhancing therapeutic efficacy while mitigating adverse effects [5]. Widely utilized DDS platforms include liposomes, biomembrane-derived vesicles, and hydrogels [6–8]. Among these, red blood cell-derived nanovesicles (RNVs) have attracted particular attention due to their intrinsic biocompatibility, extended circulation half-life, and substantial drug-loading capacity [9,10], positioning them as ideal candidates for applications in drug delivery, photothermal therapy, radiosensitization, and immune modulation [9]. Nevertheless, in oncological contexts, RNVs are predominantly sequestered by the liver rather than tumors [11,12]. This nonspecific biodistribution diminishes therapeutic efficacy and heightens the risk of hepatotoxicity, particularly in patients with preexisting hepatic impairment.

To address this challenge, surface functionalization of RNVs with tumor-targeting ligands has been investigated as a means of promoting active tumor homing [13]. Such ligands – ranging from peptides to small molecules and vitamins – bind to receptors overexpressed on cancer cells or tumor-associated endothelium. Several candidates hold considerable promise for breast cancer. The cyclic Arg-Gly-Asp (cRGD) tripeptide demonstrates high affinity for the integrin ανβ3 receptor, upregulated in both breast cancer cells and angiogenic vasculature, and has shown efficacy in enhancing nanoparticle accumulation in triple-negative breast cancer (TNBC) models [14]. Transferrin (TRF) exploits the overexpression of TRF receptors on rapidly proliferating tumor cells, a strategy validated in clinical trials with TRF-targeted liposomal oxaliplatin [15]. Folic acid (FA) targets folate receptors, which are elevated in 30–50% of breast cancers and associated with poor prognosis [16]. GE11, a 12-amino-acid peptide, binds to the epidermal growth factor receptor (EGFR), which is overexpressed in 40–70% of breast cancers, particularly TNBC and HER2-positive subtypes [17]. RVG29, derived from the rabies virus glycoprotein, binds to nicotinic acetylcholine receptors (nAChRs), thereby facilitating nanoparticle internalization and efficient traversal of the blood–brain barrier [18,19].

RNVs thus represent a highly attractive biomimetic drug delivery platform, offering biocompatibility, prolonged circulation, and immune evasion [20,21]. However, their clinical translation is hindered by insufficient tumor specificity and preferential hepatic accumulation, ultimately compromising therapeutic utility in solid tumors such as breast cancer. This study addresses a crucial gap by providing a systematic, side-by-side comparison of five tumor-targeting ligands – cRGD, TRF, FA, GE11, and RVG29 – conjugated to RNVs, an approach rarely undertaken in prior research. Existing studies have largely focused on single-ligand systems or synthetic nanoparticles, leaving a significant need for comparative evaluations of biologically derived nanocarriers. Employing a uniform lipid-anchoring strategy while controlling for vesicle size, charge, and stability, this work demonstrates distinct differences in tumor-targeting efficacy both *in vitro* and *in vivo*, with cRGD-functionalized vesicles achieving the most pronounced tumor homing in breast cancer models. Furthermore, biosafety assessments confirm negligible systemic toxicity, underscoring the translational potential of these engineered vesicles.

This study not only deepens our understanding of ligand-dependent targeting mechanisms in biomimetic nanocarriers but also establishes a critical framework for rational ligand selection in future RNV-based cancer therapeutics. Despite the availability of diverse targeting ligands, comprehensive analyses of their *in vivo* circulation dynamics and targeting efficiency remain scarce. By engineering RNVs with five distinct ligands – cRGD, TRF, FA, GE11, and RVG29 – via a lipid-anchoring strategy, and conducting rigorous comparative evaluations in a syngeneic murine breast cancer model, this work lays a robust theoretical foundation for the future development of drug-loaded RNVs and contributes meaningfully to advancing targeted nanomedicine.

## 2 Materials and methods

### 2.1 Materials

This study was conducted with the approval of the Ethics Committee of Ganzhou Hospital–Nanfang Hospital, Southern Medical University (Ethical Approval Number: TY-DKY2024-024-01). The MDA-MB-231 breast cancer cell line was obtained and maintained by the Central Laboratory of Ganzhou Hospital–Nanfang Hospital, Southern Medical University. Dulbecco’s Modified Eagle Medium (DMEM; high glucose formulation), phosphate-buffered saline (PBS), fetal bovine serum (FBS), penicillin–streptomycin, and the lipophilic membrane dye DiD were purchased from Invitrogen. Hoechst 33342 and the 4′,6-diamidino-2-phenylindole) staining kit were procured from Solarbio (Beijing, China). The targeting conjugates – 1,2-distearoyl-sn-glycero-3-phosphoethanolamine-N-polyethylene glycol 2000 (DSPE-PEG2K)-cRGD, DSPE-PEG2K-TRF, DSPE-PEG2K-FA, DSPE-PEG2K-GE11, and DSPE-PEG2K-rabies virus glycoprotein-29 (RVG29) – were obtained from Xi’an Huirui Biotechnology Co., Ltd (Xi’an, China).

### 2.2 Methods

#### 2.2.1 Preparation of RNV

Whole blood was collected from healthy mice and centrifuged at 800*g* for 5 min to isolate the red blood cell (RBC) fraction. The RBC layer was retained and washed three times with PBS to remove plasma, leukocytes, and platelets. The purified RBCs underwent three freeze–thaw cycles (−80 to 37°C) to generate RBC lysates. The lysed membranes were subsequently extruded through polycarbonate membranes with pore sizes of 800, 400, and 200 nm, ten passes each, using an Avanti mini-extruder (Advanced Thermal Solutions, Inc.). The resulting suspension was centrifuged at 20,000*g* for 1 h at 4°C to pellet the nanovesicles and eliminate free hemoglobin and cellular debris. The pellet was washed three times with PBS to ensure purity and finally re-suspended to yield RNVs.

#### 2.2.2 Preparation of RNV@cRGD, RNV@TRF, RNV@FA, RNV@GE11, and RNV@RVG29

The tumor-targeting ligands cRGD, TRF, FA, GE11, and RVG29 were incorporated onto the RNV membrane via a lipid affinity method, as previously described [21,22]. Briefly, DSPE-PEG2K-cRGD, DSPE-PEG2K-TRF, DSPE-PEG2K-FA, DSPE-PEG2K-GE11, and DSPE-PEG2K-RVG29 were dissolved by ultrasonication in a 40°C water bath for 15 min. The RNV suspension was then added to the ligand solutions and incubated with stirring at 40°C for 2 h. After cooling to room temperature, the mixture was centrifuged at 20,000*g* for 30 min at 4°C. The resulting pellets – RNV@cRGD, RNV@TRF, RNV@FA, RNV@GE11, and RNV@RVG29 – were re-suspended in PBS to obtain the final functionalized vesicles.

#### 2.2.3 Characterization of RNV and ligand-modified RNV

The average hydrodynamic diameter and zeta potential were determined using a Zetasizer Nano ZS (Malvern Instruments), while vesicle morphology was assessed by transmission electron microscopy (TEM).

##### 2.2.3.1 TEM

Samples of RNV, RNV@cRGD, RNV@TRF, RNV@FA, RNV@GE11, and RNV@RVG29 were deposited onto copper grids and allowed to stand for 30 min. Excess liquid was removed with filter paper, followed by negative staining with 3% phosphotungstic acid for 2 min. The grids were rinsed with ultrapure water, air-dried for 30 min, and subsequently imaged using TEM.

##### 2.2.3.2 Particle size, polydispersity index (PDI), and zeta potential

A Malvern particle size analyzer was employed to evaluate hydrodynamic size distribution, PDI, and zeta potential for each vesicle formulation.

##### 2.2.3.3 Stability analysis

To assess stability, particle size was monitored at 0, 1, 2, 4, 6, and 8 days post-preparation for RNV and engineered RNV formulations stored at 4 and 37°C.

#### 2.2.4 Cell culture

MDA-MB-231 breast cancer cells, cryopreserved in liquid nitrogen, were rapidly thawed at 37°C, centrifuged at 800 rpm for 3 min, and resuspended in complete medium comprising 89% DMEM high-glucose base medium, 10% FBS, and 1% penicillin–streptomycin (10,000 U/mL). Cells were seeded into culture dishes pre-equilibrated with complete medium. Cells were maintained at 37°C in a humidified incubator with 5% CO₂ and passaged every 2–3 days. Upon reaching ∼80% confluence, cells were washed twice with PBS, digested with 1 mL trypsin for 1 min, and observed microscopically for rounding and detachment. Digestion was quenched with 2 mL complete medium, and detached cells were collected by pipetting. Following centrifugation at 800 rpm for 3 min, the supernatant was discarded, and the pellet resuspended in fresh medium. Cells were subsequently reseeded at a 1:3 split ratio into new culture dishes.

#### 2.2.5 Targeting ability of RNV@cRGD, RNV@TRF, RNV@FA, RNV@GE11, and RNV@RVG29 toward breast tumor cells

According to the manufacturer’s instructions for the DiD membrane staining kit, RNV, RNV@cRGD, RNV@TRF, RNV@FA, RNV@GE11, and RNV@RVG29 were labeled with the lipophilic fluorescent probe DiD for membrane staining.

##### 2.2.5.1 Intracellular uptake experiments

MDA-MB-231 cells were cultured in confocal dishes until reaching ∼70% confluence. Equal quantities of DiD-labeled RNV, RNV@cRGD, RNV@TRF, RNV@FA, RNV@GE11, and RNV@RVG29 were added separately, while PBS served as the control. After 4 h of incubation, the supernatant was removed and cells were washed three times with PBS. Hoechst 33342 nuclear dye was added and incubated for 10 min, followed by three PBS washes. Intracellular uptake was then visualized using confocal laser scanning microscopy (CLSM; FV3000).

##### 2.2.5.2 Tumor-targeting analysis

MDA-MB-231 cells (2 × 10^5^) were seeded into six-well plates and cultured to 70% confluence. Equal quantities of DiD-labeled vesicles were added, with PBS serving as the control. Following 4 h of incubation, cells were harvested after trypsinization, washed three times with PBS, and resuspended in flow cytometry tubes for analysis using the APC fluorescence channel.

#### 2.2.6 Targeting experiments of RNV@cRGD, RNV@TRF, RNV@FA, RNV@GE11, and RNV@RVG29 in breast cancer-bearing mouse models

##### 2.2.6.1 Establishment of breast cancer xenograft model

Female BALB/c nude mice (5–6 weeks old) were acclimated for 1 week prior to modeling. MDA-MB-231 cells were expanded and harvested by trypsin digestion, washed three times with PBS, and resuspended at 2 × 10^6^ cells/100 μL. Each mouse received a subcutaneous injection into the dorsal flank. Injection sites were disinfected with alcohol prior to inoculation, after which mice were returned to cages with fresh bedding and feed.

##### 2.2.6.2 *In vivo* fluorescence imaging

Tumor-bearing nude mice were injected via the tail vein with PBS or DiD-labeled RNV, RNV@cRGD, RNV@TRF, RNV@FA, RNV@GE11, and RNV@RVG29. Whole-body fluorescence was monitored using a small animal imaging system at 0 h and subsequent time points to assess biodistribution.

##### 2.2.6.3 *Ex vivo* organ and tumor imaging

At 2 h post-injection [21], mice were euthanized by cervical dislocation. Major organs (heart, liver, spleen, lungs, kidneys) and tumor tissues were excised, arranged uniformly, and imaged to evaluate fluorescence distribution.

##### 2.2.6.4 Histological evaluation

Organs and tumors were fixed in 10% neutral formalin for 24 h, followed by alcohol dehydration, xylene clearing, and paraffin embedding. Sections were prepared and stained with hematoxylin and eosin (H&E) to assess tissue architecture and potential damage.

##### 2.2.6.5 Biosafety assessment

Tumor-bearing mice were intravenously administered PBS or DiD-labeled vesicles. After 2 h, animals were anesthetized with 1% pentobarbital and blood samples were collected via retro-orbital bleeding. Whole blood (100 μL) collected in EDTA tubes was analyzed for hematology. Serum, obtained from blood centrifuged at 2,500*g* for 15 min, was assessed for alanine aminotransferase (ALT), aspartate aminotransferase (AST), blood urea nitrogen (BUN), and creatinine (Cr) to evaluate hepatic and renal function.

##### 2.2.6.6 Enzyme-linked immunosorbent assay (ELISA) for serum proinflammatory cytokines

Serum concentrations of IL-6, TNF-α, and IL-10 in mice were quantified using ELISA kits, following the manufacturer’s protocols.

### 2.3 Statistical methods

Statistical analyses were performed using GraphPad Prism 9 (GraphPad Software, San Diego, CA). Data are presented as mean ± standard deviation (mean ± SD). The specific statistical tests used are indicated in the corresponding figure legends.


**Ethical approval:** This study was approved by the ethics committee of Ganzhou Hospital-Nanfang Hospital, Southern Medical University (Ethical Approval Number: TY-DKY2024-024-01).

## 3 Results

### 3.1 Preparation and characterization of engineered RNV

RNVs were fabricated using a sequential extrusion approach [20]. Briefly, murine RBCs were lysed to obtain membranes, which were then extruded through polycarbonate membranes with pore sizes of 800, 400, and 200 nm to generate uniform vesicles. The resulting RNVs were subsequently functionalized with targeting ligands via a lipid affinity strategy. Specifically, vesicles were incubated with five lipid-conjugated molecules: DSPE-PEG2K-cRGD, DSPE-PEG2K-TRF, DSPE-PEG2K-FA, DSPE-PEG2K-GE11, and DSPE-PEG2K-RVG29. The resulting engineered constructs (RNV@cRGD, RNV@TRF, RNV@FA, RNV@GE11, and RNV@RVG29) were subjected to comprehensive physicochemical characterization.

**Figure 1:**
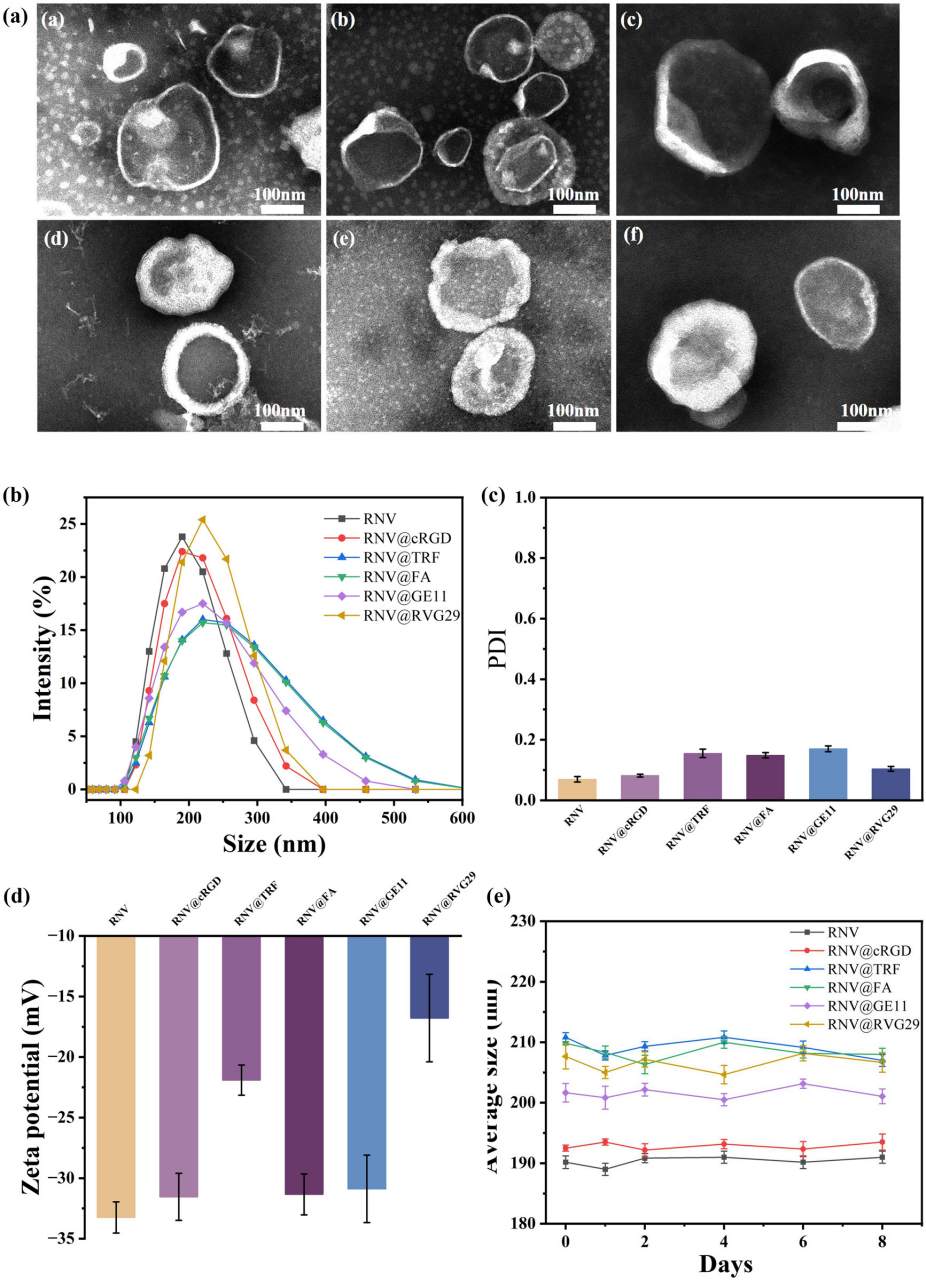
Characterization of RNV, RNV @cRGD, RNV @TRF, RNV @FA, RNV @GE11, and RNV @RVG29. (a) TEM images of (RNV (a), RNV @cRGD (b), RNV @TRF (c), RNV @FA (d), RNV @GE11 (e), and RNV @RVG29 (f)). (b) RNV particle size distribution determined by Malvern particle size analyzer. (c) PDI values of RNV particles. (d) Zeta potential characterization of RNV particles. (e) RNV particle size stability over 8 days in PBS at 4°C.

TEM revealed that both native and engineered vesicles exhibited the typical cup-shaped morphology characteristic of lipid nanovesicles (Figure 1a(a–f)). Dynamic light scattering (DLS) analysis demonstrated average diameters of 190.2 ± 1.1 nm (RNV), 192.5 ± 1.2 nm (RNV@cRGD), 210.8 ± 1.1 nm (RNV@TRF), 209.8 ± 1.5 nm (RNV@FA), 201.7 ± 1.9 nm (RNV@GE11), and 207.7 ± 2.1 nm (RNV@RVG29) (Figure 1b). As anticipated, surface modification with ligands resulted in a modest increase in particle size. The PDIs of all vesicle formulations were <0.2 (Figure 1c), confirming high sample uniformity.

Zeta potential analysis further indicated surface alterations upon ligand conjugation, with values of −33.2 ± 1.3 mV (RNV), −31.5 ± 1.9 mV (RNV@cRGD), −21.9 ± 1.2 mV (RNV@TRF), −31.3 ± 1.7 mV (RNV@FA), −30.9 ± 2.8 mV (RNV@GE11), and −16.8 ± 3.6 mV (RNV@RVG29) (Figure 1d). While RNV@cRGD, RNV@FA, and RNV@GE11 exhibited only slight decreases compared with unmodified RNV, RNV@TRF and RNV@RVG29 displayed substantial reductions in surface charge, reflecting ligand-specific physicochemical effects.

Finally, stability assessment demonstrated negligible changes in particle size across all vesicle formulations over 8 days at 4 and 37°C, confirming their structural robustness (Figures 1e and S1).

### 3.2 Tumor-cell targeting capacity of engineered RNV *in vitro*

**Figure 2:**
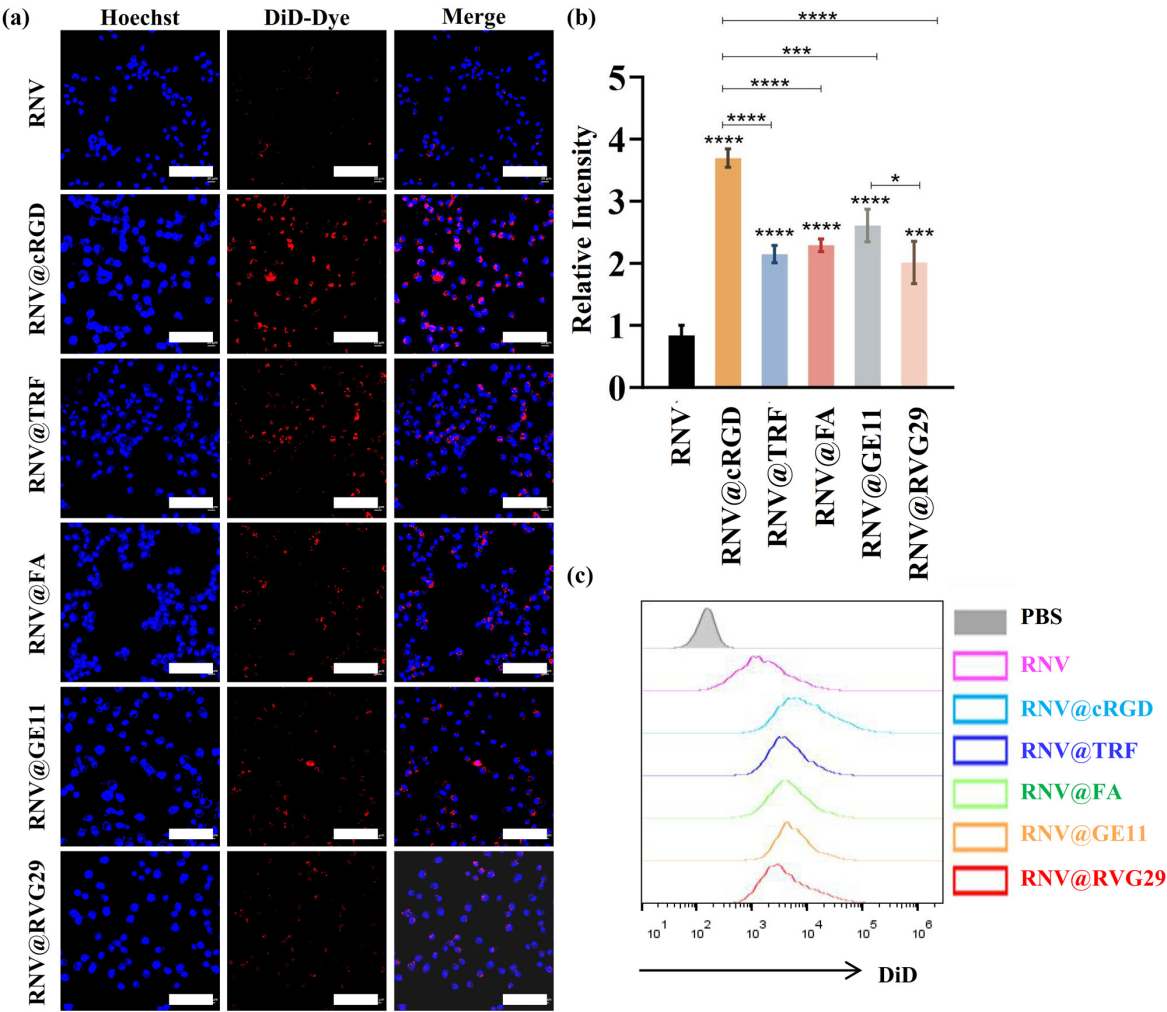
Cellular uptake of RNV and engineered RNVs by MDA-MB-231 cells. (a) CLSM images for cell uptake assay at hours after added RNV particles (scale bar = 100 μm). (b) Quantitative analysis of fluorescence intensity from CLSM imaging. (c) Flow cytometry analysis of DiD signal in MDA-MB-231 cells. Experiments were repeated at least three times, and representative images are shown. Data are shown as mean ± SD, with dots representing individual donors (average of technical duplicates). Statistical differences between groups were determined using one-way ANOVA with Tukey post-tests. *p* < 0.05 (*); *p* < 0.001 (***); *p* < 0.0001 (****); ns: not significant.

To assess the tumor-targeting efficacy of the engineered RNVs, their cellular uptake by cancer cells was first evaluated. RNV, RNV@cRGD, RNV@TRF, RNV@FA, RNV@GE11, and RNV@RVG29 were labeled with the red fluorescent membrane dye DiD and co-incubated with MDA-MB-231 breast cancer cells. As illustrated in Figure 2a, the uptake of the five engineered RNV variants by tumor cells was markedly higher than that of unmodified RNV, demonstrating that the targeting ligands enhanced nanovesicle internalization. Quantitative analysis revealed that the cRGD-modified RNV exhibited the most pronounced fluorescence signal, followed by RNV@GE11, whereas RNV@RVG29 displayed the weakest signal (Figure 2b). These observations were further corroborated by flow cytometry results (Figure 2c).

### 3.3 Tumor-targeting capacity of engineered RNV in a breast cancer bearing mice model

**Figure 3:**
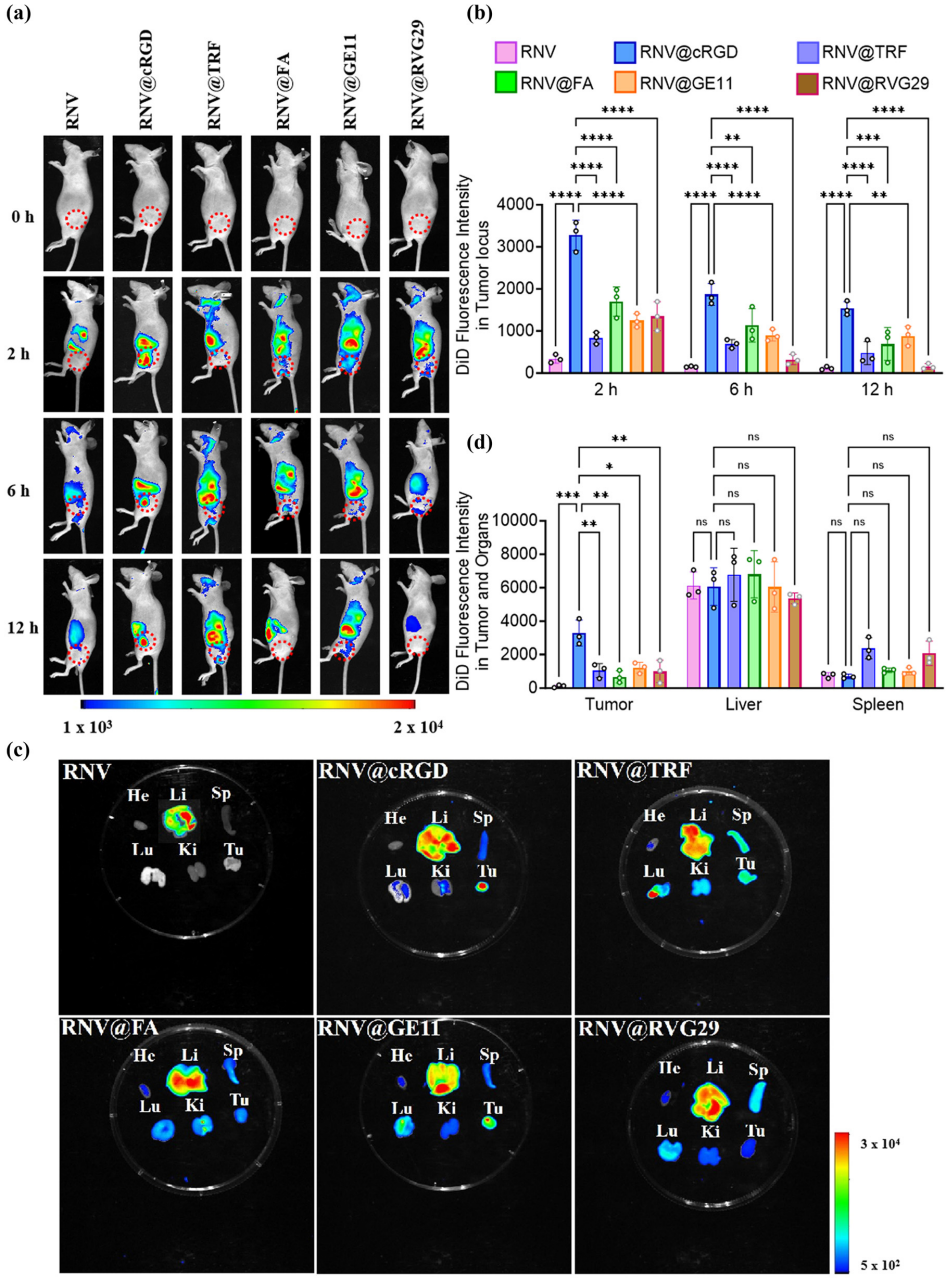
*In vivo* tumor-targeting analysis of RNV and engineered RNVs. (a) Fluorescence imaging of DiD-labeled RNV and engineered RNVs 0, 2, 6, and 12 h post-tail vein injection in MDA-MB-231 breast cancer xenograft nude mice (a: RNV; b: RNV @cRGD; c: RNV @TRF; d: RNV @FA; e: RNV @GE11; f: RNV @RVG29). (b) Quantification of fluorescence intensity across different groups and time points. (c) Distribution of RNV and engineered RNVs in major organs and tumors after 2 h post-tail vein injection (He: heart; Li: liver; Sp: spleen; Lu: lung; Ki: kidney; Tu: tumor). (d) Quantitative fluorescence intensity in organs 2 h post-injection. Data are presented as mean ± SD with individual points representing biological replicates (*N* = 3). Statistical significance was determined by one-way ANOVA with Tukey post-tests: *p* < 0.05 (*); *p* < 0.01 (**); *p* < 0.001 (***); *p* < 0.0001 (****); ns: not significant.

The tumor-targeting potential and biodistribution of the engineered RNVs were further investigated *in vivo*. MDA-MB-231 breast cancer cells were subcutaneously implanted in the dorsal region of nude mice. Upon tumor volumes reaching approximately 100 mm^3^, mice were randomly allocated into six groups: RNV, RNV@cRGD, RNV@TRF, RNV@FA, RNV@GE11, and RNV@RVG29. Pre-labeled with the red fluorescent membrane dye DiD, the RNV and engineered RNVs were administered via intravenous injection. Fluorescence imaging using the *in vivo* imaging system revealed that fluorescence signals concentrated in the tumor region as early as 2 h post-injection. Although signals diminished at 6 and 12 h, the RNV@cRGD group maintained higher fluorescence at the tumor site compared to other peptide-modified groups (Figure 3a and b), indicating superior tumor-targeting efficiency.

Subsequently, mice were euthanized by cervical dislocation, and major organs along with tumors were harvested. While unmodified RNV predominantly accumulated in the liver, engineered RNVs demonstrated varying degrees of tumor-specific accumulation. Notably, all engineered RNVs showed minor accumulation in the spleen, kidneys, and lungs, with faint fluorescence observed in the heart for RNV@TRF, RNV@FA, RNV@GE11, and RNV@RVG29 groups (Figure 3c and d). These *in vivo* biodistribution differences may stem from modifications in surface charge and composition of the engineered nanovesicles. Collectively, these results indicate that RNV@cRGD possesses superior tumor-targeting capability relative to other engineered RNVs.

### 3.4 Biosafety evaluation of engineered RNV

**Figure 4:**
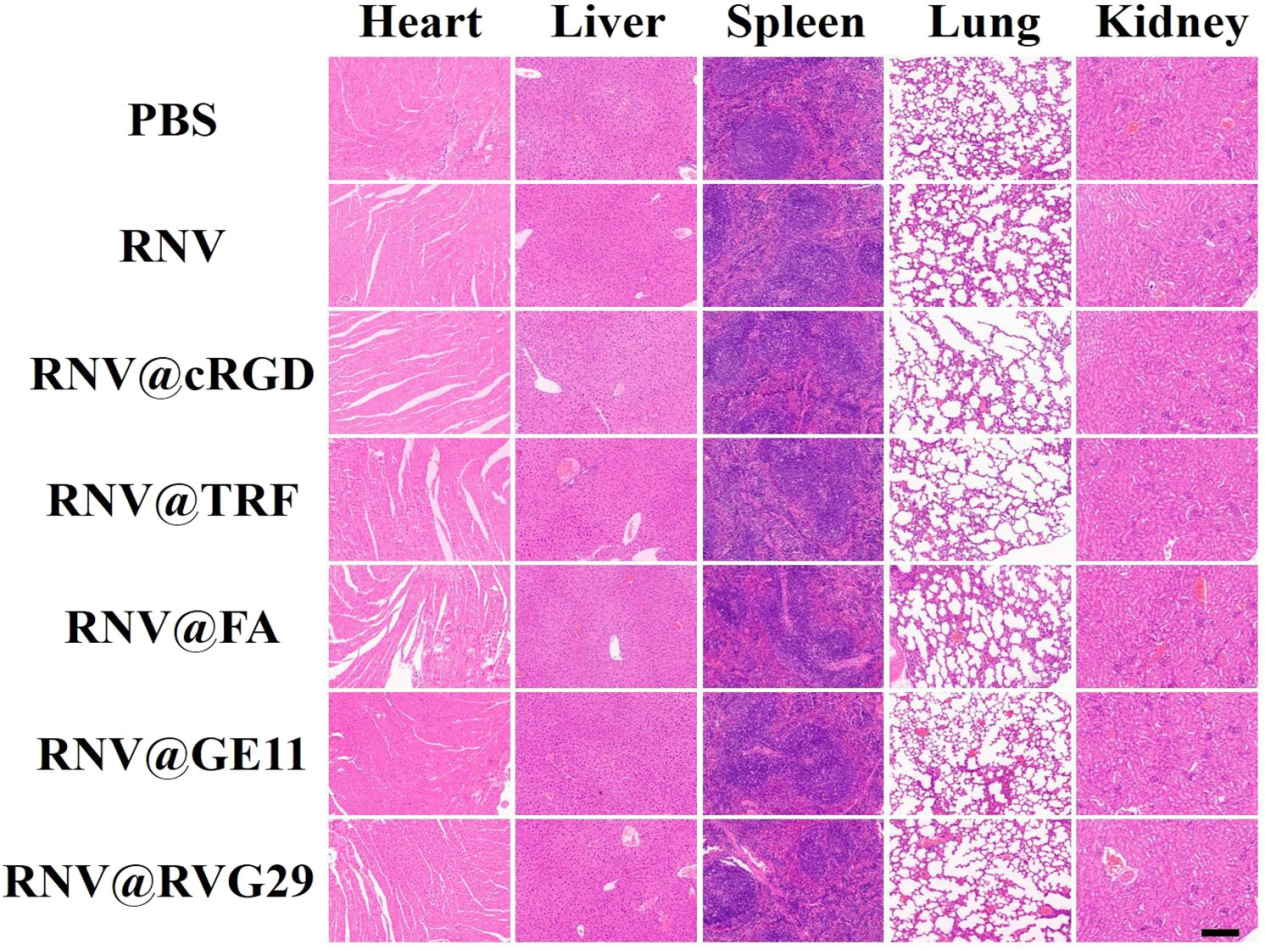
Biosafety assessment of mice following injection with RNV and engineered RNVs. H&E-stained sections of heart, liver, spleen, lung, and kidney after RNV tail injection. (200×, scale bar = 100 µm).

**Figure 5:**
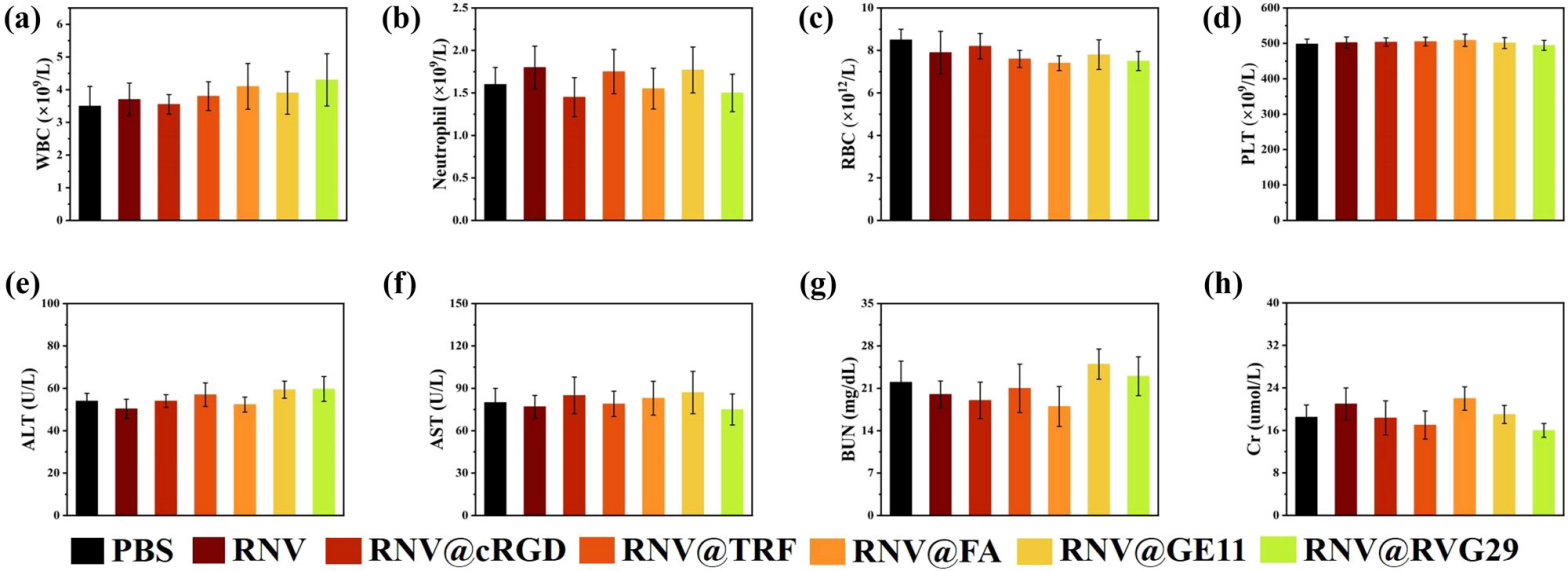
Biochemical and hematological examination after injection with RNV and engineered RNVs. White blood cell (WBC), neutrophil, RBC, platelet (PLT), ALT, AST, BUN and Cr counts in the RNV‑injected mice (a)–(h). Concentration of ALT, AST, BUN, and Cr in the mice serum (N = 3).

**Figure 6:**
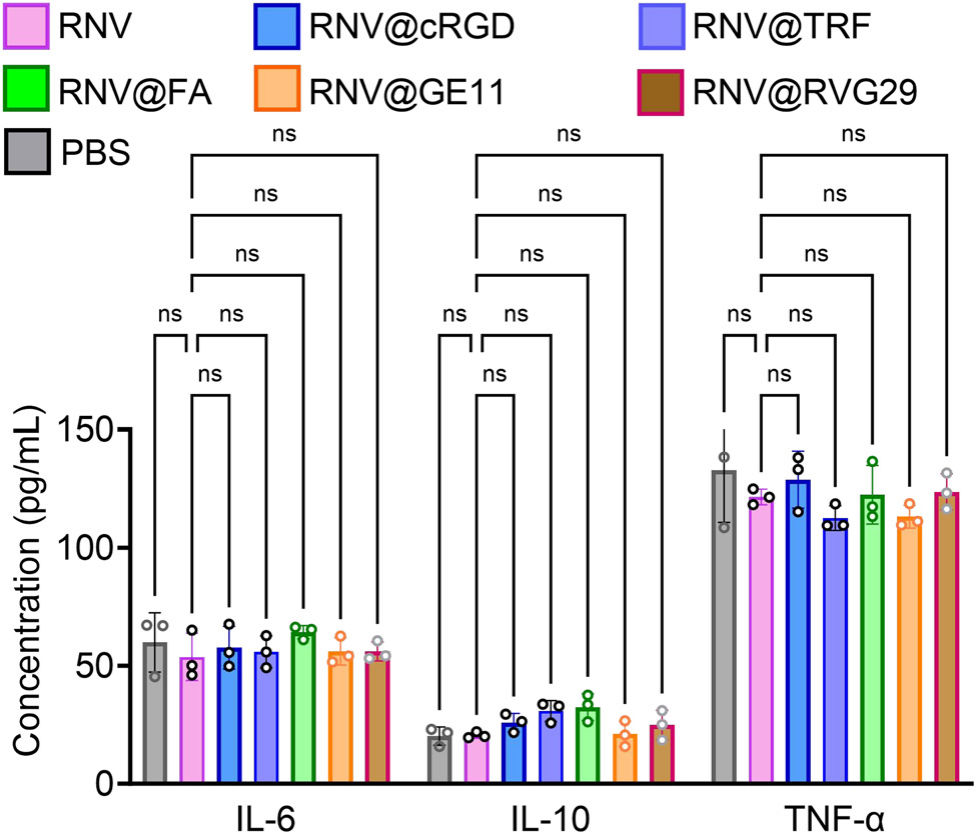
Proinflammatory cytokines expression in the serum of mice with RNV and engineered RNVs treatment. Interleukins 6 and 10 as well as TNF alpha concentration in the serum of mice after RNV and engineered RNVs. Data are presented as mean ± SD with individual points representing biological replicates (*N* = 3). Statistical significance was determined by one-way ANOVA with Tukey post-tests. ns: not significant.

The *in vivo* biosafety of the engineered RNVs was assessed. Two hours post-injection of RNV, RNV@cRGD, RNV@TRF, RNV@FA, RNV@GE11, and RNV@RVG29, nude mice were sacrificed, and major organs and tumors were collected for H&E staining. Histological examination revealed no discernible tissue damage in any treated group (Figure 4). Furthermore, biochemical, hematological analyses (Figure 5), and cytokine profile (Figure 6) demonstrated that all measured parameters remained within normal ranges, confirming that both unmodified and engineered RNVs exhibit excellent biosafety.

## 4 Discussion

With the rising incidence of breast cancer, particularly the increasing trend of early-onset cases among Chinese women, this disease has emerged as a significant threat to women’s health and survival [2]. Breast cancer is highly heterogeneous, and among its subtypes, TNBC lacks specific molecular targets, rendering conventional chemotherapy the principal therapeutic strategy [23]. However, these traditional chemotherapeutic agents are constrained by limited bioavailability, poor tumor specificity, and pronounced systemic toxicity [4]. The ongoing development of nanocarriers for drug delivery has opened new avenues for both diagnosis and therapy [24,25]. Biomimetic nanocarriers derived from cellular membranes exploit diverse biological membranes to encapsulate organic or inorganic nanoparticles, thereby constructing versatile nanocarrier systems [26].

Among these, RBC membrane-based biomimetic nanocarriers have garnered considerable attention due to their exceptional biocompatibility, prolonged circulation time, and minimal immunogenicity, suggesting broad translational potential [9,21,27]. By retaining key membrane proteins from RBCs, these nanocarriers can evade clearance by the mononuclear phagocyte system or reticuloendothelial system, thereby enhancing their passive targeting efficiency *in vivo* [28]. Nevertheless, prior studies have indicated that the inherently weak active tumor-targeting capability of RNVs limits their clinical utility in tumor imaging and therapy [13,29]. Active tumor targeting involves functionalizing nanocarriers with ligands that selectively bind to receptors on tumor cell surfaces, thereby facilitating precise drug delivery [30]. Compared to passive targeting, active targeting enhances specificity and minimizes off-target cytotoxicity [31]. The therapeutic efficacy of nanocarrier systems relies on their tumor-targeting performance: passive targeting is dictated by intrinsic biocompatibility and immunogenicity, whereas active targeting requires strategic modification with ligands [32]. Common active targeting ligands include nucleic acids, polysaccharides, small molecules, and peptides, yet systematic comparative studies on their relative efficacy remain scarce.

In this study, we first generated uniformly sized RNVs using a physical extrusion method, followed by engineering their membranes with distinct targeting ligands. TEM, DLS, and zeta potential analyses confirmed that the engineered RNVs maintained the characteristic cup-shaped morphology, exhibited increased particle size, and demonstrated reduced zeta potential. These findings indicate successful conjugation of targeting moieties onto the RNV membranes, consistent with prior studies [21,33]. The storage stability of biomimetic nanocarriers is crucial for their practical application. Our results revealed that both unmodified and engineered RNVs retained consistent particle size for over 8t days when stored in PBS at 4 and 37°C (Figure 1e), highlighting their robust stability.

We further evaluated the tumor-targeting efficacy of the engineered RNVs both *in vitro* and *in vivo*. Confocal microscopy and flow cytometry demonstrated that engineered RNVs exhibited enhanced cellular uptake relative to unmodified RNVs, with RNV@cRGD showing the most pronounced effect and RNV@RVG29 the least. *In vivo* evaluation using a breast cancer subcutaneous xenograft mouse model corroborated these findings: following intravenous administration, RNV@cRGD displayed the strongest tumor-localized fluorescence, whereas RNV@RVG29 produced relatively weaker signals. In contrast, the majority of unmodified RNVs were sequestered by the liver, with negligible tumor accumulation. Additionally, engineered RNVs demonstrated minor accumulation in the liver, kidneys, and spleen, consistent with previous observations [34,35].

According to previous studies, variations in targeting efficacy among different ligands are closely associated with the specific receptors expressed on tumor cells. The integrin αvβ3 receptor, abundantly expressed on tumor vasculature, specifically recognizes the Arg-Gly-Asp (RGD) tripeptide, with cRGD exhibiting high affinity for αvβ3. Integrin αvβ3 is prominently expressed on tumor neovascular endothelial cells as well as multiple solid tumor types, including breast cancer, glioblastoma, and osteosarcoma [36]. Our findings, consistent with prior reports, demonstrate that functionalizing RNVs with cRGD markedly enhances their tumor-targeting capability [37,38].

The rapid proliferation of tumor cells elevates their iron demand, resulting in upregulated TRF receptor expression on the surface of tumor cells, such as MDA-MB-231 and 4T1 breast cancer lines [39,40]. This overexpression allows TRF to selectively bind tumor cells, thereby augmenting the tumor-targeting efficiency of TRF-engineered RNVs. Supporting evidence includes TRF-conjugated UiO-66 metal–organic frameworks loaded with doxorubicin and indocyanine green enhanced nanocarrier tumor localization [41]. Another study employed TRF-modified hesperidin nanocarriers to induce apoptosis in TNBC cells [42]. Our *in vitro* and *in vivo* data align with these studies, confirming the improved tumor-targeting performance of TRF-modified RNVs.

FA, a stable, low-immunogenic ligand, exhibits high affinity for tumors overexpressing folate receptors, including breast and lung cancers [43]. Hanafy et al. conjugated PEGylated FA to xyloglucan-paclitaxel nanomicelles to prolong circulation and enhance uptake by folate receptor-rich breast cancer cells [44]. Furthermore, chitosan-folate-coated mesoporous silica nanoparticles carrying rAzurin inhibited tumor growth in BALB/C mice with mammary tumors. Thus, our results in line with previous indicate that FA modification enhances the tumor-targeting capability of RNVs.

The EGFR, highly expressed on numerous tumor cells including squamous cell carcinoma, breast cancer, and lung cancer, has been extensively studied and clinically exploited [6,17]. GE11, a dodecapeptide with specific EGFR affinity, has been employed to direct chitosan nanoparticles loaded with siRNA to TNBC cells, promoting apoptosis [45,46]. In this study, although EGFR is abundantly expressed on breast cancer cells, GE11 exhibited weaker tumor-targeting efficiency than cRGD, potentially due to its smaller molecular size and selective binding to a limited EGFR peptide region [47].

RVG29, a rabies virus glycoprotein-derived peptide, specifically binds nAChRs on neuronal cells and facilitates blood–brain barrier penetration, making it valuable for brain-targeted drug delivery [19]. Prior studies demonstrated that RVG29-modified PLGA nanoparticles and PEGylated organic frameworks improved central nervous system drug exposure [3,48]. Our findings indicate that RVG29 confers limited enhancement of tumor targeting in breast cancer cells, likely due to low receptor expression on MDA-MB-231 cells.

Extracellular vesicles (EVs), derived from diverse cell sources, exhibit distinct biological properties and therapeutic potentials [49–51]. Mesenchymal stem cell (MSC)-derived EVs are widely studied for their regenerative and immunomodulatory capabilities [52]. These vesicles contain anti-inflammatory cytokines, growth factors, and microRNAs that promote tissue repair, reduce fibrosis, and modulate immune responses. Clinically, MSC-EVs show promise in treating conditions like myocardial infarction, stroke, and autoimmune diseases, with advantages including low immunogenicity and reduced risk of tumorigenicity compared to cell-based therapies. In contrast, tumor cell-derived EVs harbor dual roles. While they can transfer oncogenic molecules (e.g., proteins, miRNAs) that promote cancer progression, metastasis, and immune evasion, emerging studies exploit their potential as biomarkers for early cancer detection and personalized therapy [53]. Tumor EVs also serve as vehicles for delivering therapeutic agents (e.g., siRNA, chemotherapeutics) to cancer cells, leveraging their natural targeting properties. However, their clinical application requires rigorous purification to avoid pro-tumor effects. Natural killer (NK) cell-derived EVs have emerged as potent mediators of anti-tumor immunity. These vesicles inherit cytotoxic and immunomodulatory properties from their parental NK cells, enabling them to target and eliminate cancer cells while modulating the tumor microenvironment [54–56]. Key differences lie in their biological cargo and therapeutic safety. MSC-EVs prioritize tissue repair and immune balance, whereas tumor EVs demand careful manipulation to harness their diagnostic or drug-delivery potential. Both face challenges in standardization, scalability, and long-term efficacy, but ongoing research aims to optimize their clinical translation, positioning EVs as versatile tools in regenerative medicine and oncology.

Despite these promising results, several limitations merit consideration. First, although five tumor-targeting ligands were evaluated, their selection was based predominantly on literature-reported receptor overexpression without direct experimental verification of receptor abundance or ligand–receptor binding affinities in our model. Future studies incorporating receptor profiling and quantitative ligand–receptor interaction assays, such as surface plasmon resonance or competitive binding studies, would clarify the molecular basis of targeting efficiency. Second, while differences in surface charge and composition were proposed to influence biodistribution, the mechanistic relationship between these physicochemical properties and organ-specific uptake was not directly interrogated; receptor-blocking experiments or molecular docking simulations could provide more definitive insight. Lastly, this investigation employed a single breast cancer cell line and subcutaneous xenograft model, which may not fully capture the heterogeneity or metastatic complexity of human breast cancer. Expanding the platform to orthotopic models or additional tumor types would enhance the generalizability of these findings. Nevertheless, this study provides a systematic evaluation of the influence of distinct targeting ligands on RNV tumor-targeting efficiency in breast cancer, highlighting the need for further exploration across vesicle sources and cancer types.

The findings presented herein underscore the translational potential of engineered RNVs for breast cancer therapy while also offering opportunities to enrich our understanding of immune modulation and nanomedicine design. Research on IL27RA in breast cancer highlights the role of immune checkpoint signaling in tumor progression, suggesting that ligand-engineered RNVs could concurrently deliver therapeutic agents and modulate inhibitory pathways [57]. Similarly, studies on tumor microenvironment complexity emphasize the heterogeneity of immune interactions within solid tumors, which may influence the efficacy of RNV-based delivery systems [58]. For instance, sphingolipid metabolism in immunotherapy [59] and innate lymphoid cell functions [60] have been implicated in shaping anti-tumor responses, warranting further exploration of how RNV cargo – such as immunomodulatory molecules – could interact with these pathways. Additionally, prognostic studies linking inflammatory and nutritional biomarkers to breast and colorectal cancer outcomes underscore the importance of biomarker-guided targeting approaches [61–63].

## 5 Conclusion

In this study, engineered RNVs were fabricated via an extrusion method and functionalized with five distinct targeting ligands. Both *in vitro* and *in vivo* evaluations demonstrated that these engineered RNVs markedly enhanced targeting toward breast cancer cells, with RNV@cRGD exhibiting the most pronounced effect, followed by RNV@GE11, RNV@TRF, RNV@FA, and RNV@RVG29. Furthermore, both unmodified and engineered RNVs displayed excellent biocompatibility. This work provides a systematic analysis of how distinct ligand modifications influence tumor targeting and *in vivo* biodistribution in a breast cancer model, offering valuable insights for the rational design of tumor-targeted nanotherapeutic carriers.

## Supplementary Material

Supplementary Figure

## References

[1] Chen S, Cao Z, Prettner K, Kuhn M, Yang J, Jiao L, et al. Estimates and projections of the global economic cost of 29 cancers in 204 countries and territories from 2020 to 2050. JAMA Oncol. 2023;9:465–72. 10.1001/jamaoncol.2022.7826 PMC9951101 36821107

[2] Bray F, Laversanne M, Sung H, Ferlay J, Siegel RL, Soerjomataram I, et al. Global cancer statistics 2022: GLOBOCAN estimates of incidence and mortality worldwide for 36 cancers in 185 countries. CA Cancer J Clin. 2024;74:229–63. 10.3322/caac.21834 38572751

[3] Cagel M, Grotz E, Bernabeu E, Moretton MA, Chiappetta DA. Doxorubicin: nanotechnological overviews from bench to bedside. Drug Discov Today. 2017;22:270–81. 10.1016/j.drudis.2016.11.005 27890669

[4] Baldo BA, Pham NH. Adverse reactions to targeted and non-targeted chemotherapeutic drugs with emphasis on hypersensitivity responses and the invasive metastatic switch. Cancer Metastasis Rev. 2013;32:723–61. 10.1007/s10555-013-9447-3 PMC7102343 24043487

[5] Afzal O, Altamimi ASA, Nadeem MS, Alzarea SI, Almalki WH, Tariq A, et al. Nanoparticles in drug delivery: from history to therapeutic applications. Nanomaterials (Basel). 2022;12. 10.3390/nano12244494 PMC9781272 36558344

[6] Zhao Y, Le TMD, Hong J, Jiao A, Yoon AR, Yun CO. Smart accumulating dual-targeting lipid envelopes equipping oncolytic adenovirus for enhancing cancer gene therapeutic efficacy. ACS Nano. 2024;18:27869–90. 10.1021/acsnano.4c02165 39356167

[7] Lin M, Li Y, Long H, Lin Y, Zhang Z, Zhan F, et al. Cell membrane-camouflaged DOX-loaded β-glucan nanoparticles for highly efficient cancer immunochemotherapy. Int J Biol Macromol. 2023;225:873–85. 10.1016/j.ijbiomac.2022.11.152 36402393

[8] Pan WL, Tan Y, Meng W, Huang NH, Zhao YB, Yu ZQ, et al. Microenvironment-driven sequential ferroptosis, photodynamic therapy, and chemotherapy for targeted breast cancer therapy by a cancer-cell-membrane-coated nanoscale metal-organic framework. Biomaterials. 2022;283:121449. 10.1016/j.biomaterials.2022.121449 35247637

[9] Nguyen PHD, Jayasinghe MK, Le AH, Peng B, Le MTN. Advances in drug delivery systems based on red blood cells and their membrane-derived nanoparticles. ACS Nano. 2023;17:5187–210. 10.1021/acsnano.2c11965 36896898

[10] Della Pelle G, Kostevšek N. Nucleic acid delivery with red-blood-cell-based carriers. Int J Mol Sci. 2021;22. 10.3390/ijms22105264 PMC8156122 34067699

[11] Ding L, Wu Y, Wu M, Zhao Q, Li H, Liu J, et al. Engineered red blood cell biomimetic nanovesicle with oxygen self-supply for near-infrared-II fluorescence-guided synergetic chemo-photodynamic therapy against hypoxic tumors. ACS Appl Mater Interfaces. 2021;13:52435–49. 10.1021/acsami.1c19096 34705421

[12] Zheng D, Yu P, Wei Z, Zhong C, Wu M, Liu X. RBC membrane camouflaged semiconducting polymer nanoparticles for near-infrared photoacoustic imaging and photothermal therapy. Nanomicro Lett. 2020;12:94. 10.1007/s40820-020-00429-x PMC7770914 34138120

[13] Su J, Sun H, Meng Q, Yin Q, Tang S, Zhang P, et al. Long circulation red-blood-cell-mimetic nanoparticles with peptide-enhanced tumor penetration for simultaneously inhibiting growth and lung metastasis of breast cancer. Adv Funct Mater. 2016;26:1243–52.

[14] Yuan Z, Gui L, Zheng J, Chen Y, Qu S, Shen Y, et al. GSH-activated light-up near-infrared fluorescent probe with high affinity to α(v)β(3) integrin for precise early tumor identification. ACS Appl Mater Interfaces. 2018;10:30994–1007. 10.1021/acsami.8b09841 30141897

[15] Mojarad-Jabali S, Mahdinloo S, Farshbaf M, Sarfraz M, Fatahi Y, Atyabi F, et al. Transferrin receptor-mediated liposomal drug delivery: recent trends in targeted therapy of cancer. Expert Opin Drug Deliv. 2022;19:685–705. 10.1080/17425247.2022.2083106 35698794

[16] Mohammadi Barzelighi H, Bakhshi B, Daraei B, Mirzaei A. Investigating the effect of rAzurin loaded mesoporous silica nanoparticles enwrapped with chitosan-folic acid on breast tumor regression in BALB/(C) mice. Int J Biol Macromol. 2025;300:139245. 10.1016/j.ijbiomac.2024.139245 39732269

[17] Hailing T, Yonghong P, Yufeng Z, Haitao T. Challenges for the application of EGFR-targeting peptide GE11 in tumor diagnosis and treatment. J Control Rel. 2022;349:592–605. 10.1016/j.jconrel.2022.07.018 35872181

[18] Yang Q, Li R, Hong Y, Liu H, Jian C, Zhao S. Curcumin-loaded gelatin nanoparticles cross the blood–brain barrier to treat ischemic stroke by attenuating oxidative stress and neuroinflammation. Int J Nanomed. 2024;19:11633–49. 10.2147/IJN.S487628 PMC11568047 39553455

[19] Oswald M, Geissler S, Goepferich A. Targeting the central nervous system (CNS): a review of rabies virus-targeting strategies. Mol Pharm. 2017;14:2177–96. 10.1021/acs.molpharmaceut.7b00158 28514853

[20] Wang X, Mao K, Zhang X, Zhang Y, Yang Y-G, Sun T. Red blood cell derived nanocarrier drug delivery system: a promising strategy for tumor therapy. Interdiscip Med. 2024;2:e20240014.

[21] Nie C, Pan W, Wu B, Luo T, Lv J, Fan Y, et al. Engineered enzyme-loaded erythrocyte vesicles precisely deprive tumoral nutrients to induce synergistic near-infrared-II photothermal therapy and immune activation. ACS Nano. 2023;17:13211–23. 10.1021/acsnano.3c00345 37440429

[22] Zhu Q, Ling X, Yang Y, Zhang J, Li Q, Niu X, et al. Embryonic stem cells-derived exosomes endowed with targeting properties as chemotherapeutics delivery vehicles for glioblastoma therapy. Adv Sci (Weinh). 2019;6:1801899. 10.1002/advs.201801899 PMC6425428 30937268

[23] Bianchini G, Balko JM, Mayer IA, Sanders ME, Gianni L. Triple-negative breast cancer: challenges and opportunities of a heterogeneous disease. Nat Rev Clin Oncol. 2016;13:674–90. 10.1038/nrclinonc.2016.66 PMC5461122 27184417

[24] Chen M, Sun Y, Liu HY. Cell membrane biomimetic nanomedicines for cancer phototherapy. Interdiscip Med. 2023;1:e20220012.

[25] Zhang L, Ma W, Gan X, Chen W, Guo J, Cui Y, et al. Adequate enrichment of extracellular vesicles in laboratory medicine. Interdiscip Med. 2023;1:e20220003.

[26] Zhou J, Kroll AV, Holay M, Fang RH, Zhang L. Biomimetic nanotechnology toward personalized vaccines. Adv Mater. 2020;32:e1901255. 10.1002/adma.201901255 PMC6918015 31206841

[27] Vincy A, Mazumder S, Amrita, Banerjee I, Hwang KC, Vankayala R. Recent progress in red blood cells-derived particles as novel bioinspired drug delivery systems: challenges and strategies for clinical translation. Front Chem. 2022;10:905256. 10.3389/fchem.2022.905256 PMC9092017 35572105

[28] Yang S, He B, He C, Zhao F, Li R, Shi M, et al. Red blood cell membrane-camouflaged reduction-responsive polyethylenimine-based nanoparticles for enhanced antitumor efficacy of antisense oligonucleotides. Mol Pharm. 2025. 10.1021/acs.molpharmaceut.5c00412 40726257

[29] Chen S, Fan J, Xiao F, Qin Y, Long Y, Yuan L, et al. Erythrocyte membrane-camouflaged Prussian blue nanocomplexes for combinational therapy of triple-negative breast cancer. J Mater Chem B. 2023;11:2219–33. 10.1039/d2tb02289c 36790882

[30] Liu Y, Hu J, Shu Y, Li G, Jin F, Ren J, et al. Engineered red blood cell extracellular vesicles for delivery of Dox and siIDO1 enhance targeted chemo-immunotherapy of acute myeloid leukemia. J Immunother Cancer. 2025;13. 10.1136/jitc-2024-011148 PMC12265820 40664450

[31] Cai N, Zhan X, Zhang Q, Di H, Chen C, Hu Y, et al. Red blood cell-derived small extracellular vesicles inhibit influenza virus through surface-displayed sialic acids. Angew Chem Int Ed Engl. 2025;64:e202413946. 10.1002/anie.202413946 39275883

[32] Ding J, Ding X, Liao W, Lu Z. Red blood cell-derived materials for cancer therapy: construction, distribution, and applications. Mater Today Bio. 2024;24:100913. 10.1016/j.mtbio.2023.100913 PMC10767221 38188647

[33] Peng B, Yang Y, Wu Z, Tan R, Pham TT, Yeo EYM, et al. Red blood cell extracellular vesicles deliver therapeutic siRNAs to skeletal muscles for treatment of cancer cachexia. Mol Ther. 2023;31:1418–36. 10.1016/j.ymthe.2023.03.036 PMC10188904 37016578

[34] Chiangjong W, Panachan J, Keadsanti S, Newburg DS, Morrow AL, Hongeng S, et al. Development of red blood cell-derived extracellular particles as a biocompatible nanocarrier of microRNA-204 (REP-204) to harness anti-neuroblastoma effect. Nanomedicine. 2024;60:102760. 10.1016/j.nano.2024.102760 38852882

[35] Xia Q, Zhang Y, Li Z, Hou X, Feng N. Red blood cell membrane-camouflaged nanoparticles: a novel drug delivery system for antitumor application. Acta Pharm Sin B. 2019;9:675–89. 10.1016/j.apsb.2019.01.011 PMC6663920 31384529

[36] Huang X, Wu W, Jing D, Yang L, Guo H, Wang L, et al. Engineered exosome as targeted lncRNA MEG3 delivery vehicles for osteosarcoma therapy. J Control Rel. 2022;343:107–17. 10.1016/j.jconrel.2022.01.026 35077741

[37] Xu J, Li R, Yan D, Zhu L. Biomimetic modification of siRNA/chemo drug nanoassemblies for targeted combination therapy in breast cancer. ACS Appl Mater Interfaces. 2024;16:59765–76. 10.1021/acsami.4c11064 39447113

[38] Wu W, Guo H, Jing D, Zhang Z, Zhang Z, Pu F, et al. Targeted delivery of PD-L1-derived phosphorylation-mimicking peptides by engineered biomimetic nanovesicles to enhance osteosarcoma treatment. Adv Healthc Mater. 2022;11:e2200955. 10.1002/adhm.202200955 PMC11468027 36123781

[39] Kulkarni S, Soman S, Pandey A, Mutalik S. Engineered transferrin-conjugated PEGylated multifunctional MOF-74 as precision nanotheranostics for triple-negative breast cancer. Int J Biol Macromol. 2025;322:146857. 10.1016/j.ijbiomac.2025.146857 40816394

[40] Yu X, Cheng L, Liu S, Wang M, Zhang H, Wang X, et al. Correlation between ferroptosis and adriamycin resistance in breast cancer regulated by transferrin receptor and its molecular mechanism. Faseb J. 2024;38:e23550. 10.1096/fj.202302597R 38466338

[41] Soman S, Kulkarni S, John J, Vineeth P, Ahmad SF, George SD, et al. Transferrin-conjugated UiO-66 metal organic frameworks loaded with doxorubicin and indocyanine green: a multimodal nanoplatform for chemo-photothermal-photodynamic approach in cancer management. Int J Pharm. 2024;665:124665. 10.1016/j.ijpharm.2024.124665 39236772

[42] Giri D, Dey SK, Manna S, Das Chaudhuri A, Mahata R, Pradhan A, et al. Nanoconjugate carrying pH-responsive transferrin receptor-targeted hesperetin triggers triple-negative breast cancer cell death through oxidative attack and assemblage of pro-apoptotic proteins. ACS Appl Bio Mater. 2024;7:7556–73. 10.1021/acsabm.4c01131 39504304

[43] Song DG, Ye Q, Poussin M, Chacon JA, Figini M, Powell DJ, Jr.. Effective adoptive immunotherapy of triple-negative breast cancer by folate receptor-alpha redirected CAR T cells is influenced by surface antigen expression level. J Hematol Oncol. 2016;9:56. 10.1186/s13045-016-0285-y PMC4955216 27439908

[44] Boogerd LS, Boonstra MC, Beck AJ, Charehbili A, Hoogstins CE, Prevoo HA, et al. Concordance of folate receptor-α expression between biopsy, primary tumor and metastasis in breast cancer and lung cancer patients. Oncotarget. 2016;7:17442–54. 10.18632/oncotarget.7856 PMC4951224 26943581

[45] Abdulmalek SA, Saleh AM, Shahin YR, El Azab EF. Functionalized siRNA-chitosan nanoformulations promote triple-negative breast cancer cell death via blocking the miRNA-21/AKT/ERK signaling axis: in-silico and in vitro studies. Naunyn Schmiedebergs Arch Pharmacol. 2024;397:6941–62. 10.1007/s00210-024-03068-w PMC11422444 38592437

[46] Gu H, Shi R, Xu C, Lv W, Hu X, Xu C, et al. EGFR-targeted liposomes combined with ginsenoside Rh2 inhibit triple-negative breast cancer growth and metastasis. Bioconjug Chem. 2023;34:1157–65. 10.1021/acs.bioconjchem.3c00207 37235785

[47] Ruoslahti E. Peptides as targeting elements and tissue penetration devices for nanoparticles. Adv Mater. 2012;24:3747–56. 10.1002/adma.201200454 PMC3947925 22550056

[48] Yan T, Liao Q, Chen Z, Xu Y, Zhu W, Hu P, et al. β-Ketoenamine covalent organic framework nanoplatform combined with immune checkpoint blockade via photodynamic immunotherapy inhibit glioblastoma progression. Bioact Mater. 2025;44:531–43. 10.1016/j.bioactmat.2024.10.029 PMC11583667 39584065

[49] Welsh JA, Goberdhan DCI, O’driscoll L, Buzas EI, Blenkiron C, Bussolati B, et al. Minimal information for studies of extracellular vesicles (MISEV2023): from basic to advanced approaches. J Extracell Vesicles. 2024;13:e12404. 10.1002/jev2.12404 PMC10850029 38326288

[50] Wang J, Yuan S, Tu Y, Lv Z, Cheng H, Ding X. Extracellular vesicles in skin health, diseases, and aging. Interdiscip Med. 2024;2:e20240011.

[51] Li Y, Zhang H, Jiang Y, Yang J, Cai D, Bai X. The application of extracellular vesicles in orthopedic diseases. Interdiscip Med. 2024;2:e20230055.

[52] Lu T, Zhang J, Cai J, Xiao J, Sui X, Yuan X, et al. Extracellular vesicles derived from mesenchymal stromal cells as nanotherapeutics for liver ischaemia-reperfusion injury by transferring mitochondria to modulate the formation of neutrophil extracellular traps. Biomaterials. 2022;284:121486. 10.1016/j.biomaterials.2022.121486 35447404

[53] Xie F, Zhou X, Su P, Li H, Tu Y, Du J, et al. Breast cancer cell-derived extracellular vesicles promote CD8(+) T cell exhaustion via TGF-β type II receptor signaling. Nat Commun. 2022;13:4461. 10.1038/s41467-022-31250-2 PMC9343611 35915084

[54] Yue X, Cui J, Ren S, Zhang Y, Li Y, Cui H, et al. NK-cell-derived extracellular vesicles engineered to carry senolytics eliminate chemotherapy-induced senescent osteosarcoma cells. J Extracell Vesicles. 2025;14:e70123. 10.1002/jev2.70123 PMC12281467 40693561

[55] Wu N, Yang N, Zhang S, Wu H, Fang X, Lin W, et al. Chimeric antigen receptor NK cells for breast cancer immunotherapy. Cancer Treat Rev. 2025;137:102943. 10.1016/j.ctrv.2025.102943 40305951

[56] Gong Y, Klein Wolterink RGJ, Wang J, Bos GMJ, Germeraad WTV. Chimeric antigen receptor natural killer (CAR-NK) cell design and engineering for cancer therapy. J Hematol Oncol. 2021;14:73. 10.1186/s13045-021-01083-5 PMC8088725 33933160

[57] Chen Y, Anwar M, Wang X, Zhang B, Ma B. Integrative transcriptomic and single-cell analysis reveals IL27RA as a key immune regulator and therapeutic indicator in breast cancer. Discov Oncol. 2025;16:977. 10.1007/s12672-025-02811-w PMC12127260 40450602

[58] Li Z, Li J, Bai X, Huang X, Wang Q. Tumor microenvironment as a complex milieu driving cancer progression: a mini review. Clin Transl Oncol. 2025;27:1943–52. 10.1007/s12094-024-03697-w PMC12033186 39342061

[59] Wang Q, Zheng C, Hou H, Bao X, Tai H, Huang X, et al. Interplay of sphingolipid metabolism in predicting prognosis of GBM patients: towards precision immunotherapy. J Cancer. 2024;15:275–92. 10.7150/jca.89338 PMC10751665 38164288

[60] Shen G, Wang Q, Li Z, Xie J, Han X, Wei Z, et al. Bridging chronic inflammation and digestive cancer: the critical role of innate lymphoid cells in tumor microenvironments. Int J Biol Sci. 2024;20:4799–818. 10.7150/ijbs.96338 PMC11414386 39309440

[61] Wang K, Li K, Zhang Z, Zeng X, Sulayman S, Ababaike S, et al. Prognostic value of combined NP and LHb index with absolute monocyte count in colorectal cancer patients. Sci Rep. 2025;15:8902. 10.1038/s41598-025-94126-7 PMC11909193 40087531

[62] Li K, Zeng X, Zhang Z, Wang K, Pan Y, Wu Z, et al. Pan‑immune‑inflammatory values predict survival in patients after radical surgery for non‑metastatic colorectal cancer: a retrospective study. Oncol Lett. 2025;29:197. 10.3892/ol.2025.14943 PMC11880885 40046636

[63] Chen Y, Zhang B, Wang X, Chen Y, Anwar M, Fan J, et al. Prognostic value of preoperative modified Glasgow prognostic score in predicting overall survival in breast cancer patients: a retrospective cohort study. Oncol Lett. 2025;29:180. 10.3892/ol.2025.14926 PMC11843409 39990808

