# Supplementary figures and images for: Engineered RBC-derived nanovesicles functionalized with tumor-targeting ligands: A comparative study on breast cancer targeting efficiency and biocompatibility
Source: Open Med (Wars). 2025 Oct 31;20(1):20251306. doi: 10.1515/med-2025-1306 (PMC12596866; doi:10.1515/med-2025-1306)

# Supplementary material

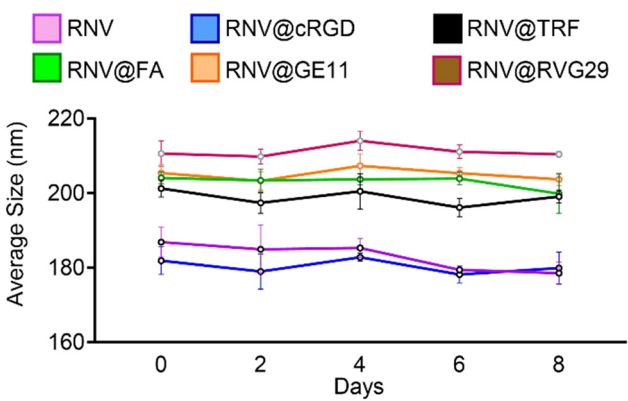

Figure S1: RNV particle size stability over 8 days in PBS at 37°C.

Supplement: Supplementary Figure [file med-2025-1306-sm.pdf]
